# Recombinant mussel proximal thread matrix protein promotes osteoblast cell adhesion and proliferation

**DOI:** 10.1186/s12896-016-0247-z

**Published:** 2016-02-16

**Authors:** Hee Young Yoo, Young Hoon Song, Mathias Foo, Eunseok Seo, Dong Soo Hwang, Jeong Hyun Seo

**Affiliations:** Division of Integrative Biosciences and Biotechnology, Pohang University of Science and Technology, Pohang, 790-784 Korea; School of Chemical Engineering, Yeungnam University, Gyeongsan, 712-749 Korea; School of Engineering, University of Warwick, Coventry, CV4 7AL UK; School of Environmental Science and Engineering, Pohang University of Science and Technology, Pohang, 790-784 Korea

**Keywords:** PTMP1, Cell adhesion, vWF, Mussel adhesion, Cell adhesion, *Escherichia coli*

## Abstract

**Background:**

von Willebrand factor (VWF) is a key load bearing domain for mamalian cell adhesion by binding various macromolecular ligands in extracellular matrix such as, collagens, elastin, and glycosaminoglycans. Interestingly, vWF like domains are also commonly found in load bearing systems of marine organisms such as in underwater adhesive of mussel and sea star, and nacre of marine abalone, and play a critical load bearing function. Recently, Proximal Thread Matrix Protein1 (PTMP1) in mussel composed of two vWF type A like domains has characterized and it is known to bind both mussel collagens and mammalian collagens.

**Results:**

Here, we cloned and mass produced a recombinant PTMP1 from *E. coli* system after switching all the minor codons to the major codons of *E. coli*. Recombinant PTMP1 has an ability to enhance mouse osteoblast cell adhesion, spreading, and cell proliferation. In addition, PTMP1 showed vWF-like properties as promoting collagen expression as well as binding to collagen type I, subsequently enhanced cell viability. Consequently, we found that recombinant PTMP1 acts as a vWF domain by mediating cell adhesion, spreading, proliferation, and formation of actin cytoskeleton.

**Conclusions:**

This study suggests that both mammalian cell adhesion and marine underwater adhesion exploits a strong vWF-collagen interaction for successful wet adhesion. In addition, vWF like domains containing proteins including PTMP1 have a great potential for tissue engineering and the development of biomedical adhesives as a component for extra-cellular matrix.

## Background

In order for mussels to live and adapt in an environment that has mechanical challenges due to waves and currents, they are able to attach themselves to the surface in both dry and wet states through byssus. The byssus is an outstanding material with a unique holdfast structure that is composed of individual threads rooted from mussel stem. The byssus threads are mostly composed of proteins and each of the threads has three different parts, i.e. a flexible and elastic proximal part, a rigid and stiff distal part and an adhesive plaque at the end [1, 2]. The change of the mechanical properties of the byssus thread is not sudden but gradual due to the mechanical gradient of thread collagenous protein called preCols [3]. PreCols are block copolymer like proteins, which have central collagenous domain that is flanked with two silk-like domains which is prepepsinized distal collagen (preCol-D), the elastin-like domain prepepsinized proximal collagen (preCol-P), or the glycine and asparagine rich domaine prepepsinized collagen (preCol-NG), and terminated by histidine-rich repeat sequences [4]. The distal portion of the byssus is dominated by the densely packed collagen bundles, while the proximal portion of the byssus has only 30 % of collagen and the rest is the matrix protein, which enables the proximal portion of the byssus for cyclic stress at a fixed strain. The proximal thread has prominent strain stiffening property while the distal thread has stress softening property in which the matrix protein plays an important role in mediating the different mechanical properties between the proximal and the distal threads [2, 5]. There are two different types of byssal matrix proteins that are identified, namely the Thread Matrix Proteins (TMPs) and the Proximal Thread Matrix Protein1 (PTMP1) [6, 7].

**Fig. 1:**
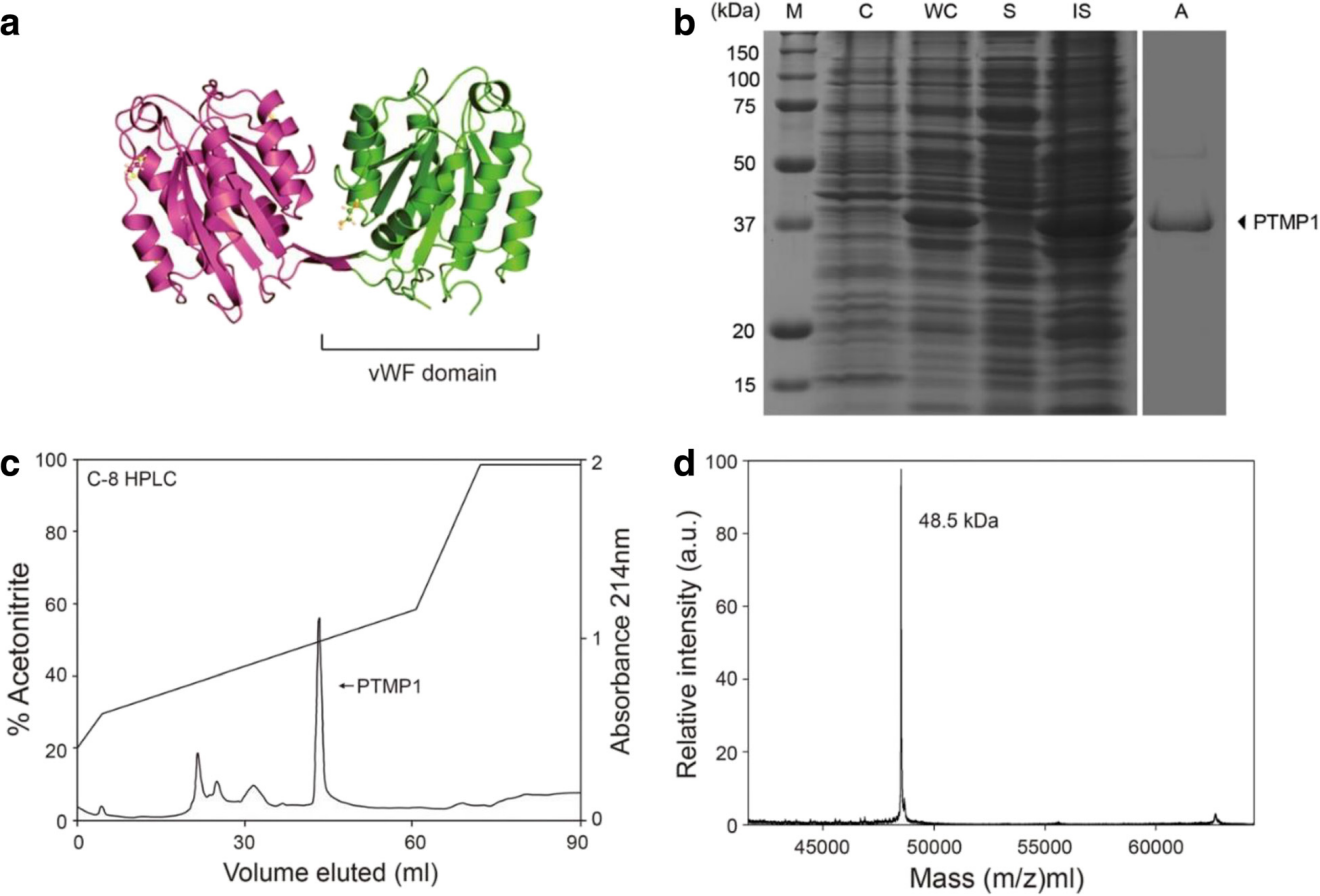
**a** PTMP1 structure from PDB (http://www.rcsb.org) **b** SDS-PAGE analysis of recombinant PTMP1. Lanes: M, protein maker; WC, whole cell sample; S, soluble fraction; IS, insoluble fraction; A, purified PTMP1 protein. **c** HPLC purification of recombinant PTMP1 with a reverse phase column chromatography and **d** MALDI-TOF analysis of a purified recombinant PTMP1

Recently, PTMP1 which is preferentially located in the proximal portion than the distal portion of each byssal thread was purified and crystallized from *M. galloprovincialis* and its protein structure had been revealed (Fig. 1a) [7, 8]. According to the structure, PTMP1 has the molecular weight of 48.5 kDa and the protein upon moderately post-translational modified by glycans, was observed to have two sequence stretches with homology to the group of von Willebrand factor type A (vWF) domains [8, 9]. vWF is a large multimeric glycoprotein presented in blood plasma, endothelial cells, and subendothelial matrix of the vessel. vWF is widely distributed as a functional domain in other extracellular protein superfamilies including integrins which are involved in binding various macromolecular ligands for instance collagens, laminins, and glycosaminoglycans such as heparin and hyaluronan. vWF has four types of domain, i.e. domains A, B, C, and D. It has been recently shown that the structure of PTMP1 has 50 % homology to vWF A domain of the integrins [8, 10].

As mentioned earlier, integrins have been known as major cell receptor that binds with fibronectins and laminins and they activate several intracellular signaling pathways to control cellular processes. As the vWF A domain in integrins binds to specific peptides, they form clusters in the cell membrane and reorganize actin filaments into larger stress fibers. The clusters formed by integrins in the cell membrane result in the association of a signaling complex that regulates cell proliferation [11–14].

Here, the recombinant PTMP1 that is known to have a homology of the integrins vWF domain are mass produced from *E. coli*. PTMP1 mediates the attachment of mouse pre-osteoblast cells (MC-3 T3) to collagen matrix, as well as leading the cells to anchor to the solid substratum, thereby enhancing cell proliferation.

## Methods

### Constructions of strains and plasmid

A recombinant proximal thread matrix protein (PTMP1) was synthesized after switching all the minor codons to the major codons of *E. coli* and cloned into pET28(+) vector (Novagen, Merck, Whitehouse Station, NJ, USA) utilizing the following restriction enzyme sites: *Nde* I and *Hind* III. The cloned vector was verified to contain PTMP1 sequence through DNA sequencing. To express this recombinant proteins, the vector was then inserted into the host strain *E. coli* [BL21(DE3)] [F- ompT hsdSB (rB- mB-) gal dcm ∆(srl-recA) 306::Tn10(DE3)] (Novagen).

### Conditions of media and cell culture

To construct and produce the recombinant PTMP1 strain and expression, the host *E. coli* cells were grown in Luria-Bertani (LB) medium and this constructed transformant containing the recombinant plasmid was stored at −80 °C. The cultures were carried out in LB medium with 50 μg/mL Kanamycine (Sigma, St. Louis, USA) added to act as a selection marker in 10 L Bioreactor (Kobiotech) at 37 °C and 250 rpm. With the optical density at 600 nm, an ultraviolet (UV)-visible spectrophotometer (OPTIZEN POP, Mecasys, Daejeon, S. Korea) was used to monitor cell growth. As the cultures attained an OD_600_ of ~0.4, the final concentration of 1 mM isopropyl-β-D-thiogalactopyranoside (IPTG) was added to the culture broth for the induction of PTMP1 production. The samples were collected 9 h after the induction. Then, the samples is centrifuged for 10 min at 4 °C at 18,000 g prior storing the cell pallets at −80 °C for further analysis.

### Purification of PTMP1

The 5 ml lysis buffer (10 mM Tris-Cl, 100 mM sodium phosphate, pH 8.0) is used to resuspend the harvested cell pellets per gram wet weight. Through the use of constant cell disruption systems (Constant Systems), the samples were lysed at 20 kPsi before they were centrifuged for 20 min at 4 °C at 18,000 g. The debris of the cell was gathered for purification. To extract the PTMP1, the cell lysate pellet was resuspended in 10 % (v/v) acetic acid and the extracting solution was centrifuged for 20 min at 4 °C at 18,000 g. Using Spectra/Por molecular porous membrane tubing (MWCO 14,000 Da, Spectrum Laboratories, USA), the supernatant was collected and dialyzed at 4 °C in 1 % (v/v) acetic acid buffer overnight. Through freeze-drying process, the purified sample was concentrated, which will be used for further purification. The concentrated PTMP1 was injected to Aquapore RP-300 column (C8, 250 × 7.0 mm, Brownlee, Perkin-Elmer, USA) at the flow rate of 1.0 mL/min and following the same gradient condition as the previous PTMP1 purification method from mussels [15].

### SDS-PAGE and amino acid analysis

The purity of PTMP1 (~95 %) was verified through the use SDS-PAGE and amino acid analysis. In the protein sample buffer (0.5 M Tris–HCl (pH 6.8), 10 % glycerol, 5 % sodium dodecyl sulfate (SDS), 5 % β-mercaptoethanol, 0.25 % bromophenol blue), the samples were resuspended and then heated to 100 °C for 5 min before they were centrifuged for 1 min. Then, using 12 % (w/v) SDS-polyacrylamide gel electrophoresis (PAGE) and Coomassie-blue staining (Bio-Rad), the samples were separated and detected respectively. System S4300 Amino Acid Analyzer (SYKAM, Germany) was used for the determination of the total protein concentration and purity.

### MALDI-TOF MS analysis

The 4700 Proteomics Analyzer (Applied Biosystems, Thermo, USA) was used to perform matrix-assisted laser desorption ionization mass spectrometry with time-of-flight (MALDI-ToF/MS) analysis in the positive ion linear mode, where the used matrix solutions were Sinapinic acid in 30 % acetonitrile and 0.1 % trifluoroacetic. With this matrix solution, the samples were diluted at 1:25 ratio. The amount of 1 ml of the mixture was spotted onto the MALDI sample target plates before evaporated with a vacuum pump. The obtained spectra were between the mass range of 20,000 Da and 70,000 Da with 1500 laser shots.

### Mammalian cell proliferation assays

With the use of 10 % (v/v) fetal bovine serum (FBS; Hyclone) and penicillin/streptomycin (Hyclone), the MC3T3-E1 (RIKEN Cell Bank, Japan) mouse preosteoblast cells were cultured in alpha-minimal essential medium (α-MEM; Hyclone) at 37 °C in a humidified atmosphere with 5 and 95 % CO_2_ and air respectively. All cells were collected by trypsinization. Then, the cells were washed twice in α-MEM before they were diluted to an approximate concentration of 1 × 10^5^ cells per 1 mL of α-MEM without FBS. In serum-free medium, a total amount of 50,000 cells with 95 % viability were placed on each untreated polystryrene 24-well culture plates (SPL Life Science), where these wells were coated with PTMP1. The uncoated wells were used as negative controls. The amount of coating material used was 3.5 mg per cm^2^ of well area. These wells were coated under room temperature in pH7.4 PBS buffer overnight. To quantify the measure of cell number after cell attachment and proliferation, the Cell Counting Kit-8 (CCK-8; Dojindo Laboratories), which contains 2-(2-methoxy-4-nitrophenyl)-3-(4-nitrophenyl)-5-(2,4-disulfophenyl)-2H-tetrazolium (WST-8) and produces a formazan dye that is highly water soluble upon the reduction in the presence of an electron carrier was used. At a given time, the CCK-8 assay was performed in an appropriate manner, i.e., after every 24 h during a 72 h incubation in serum-containing α-MEM. A total amount of 25 μL of the CCK-8 solution was added to each well. Then, these plates were further incubated at 37 °C for 3 h. After the incubation, a microplate absorbance spectrophotometer (Bio-Rad, USA) is used to measure the absorbance at 450 nm.

### Identification of type I collagen expression through RT-PCR analysis of osteoblastic MC3T3-E1 cells mRNA

The expression of collagen type I (Col I) mRNA in osteoblastic MC3T3-E1 cells was investigated by performing reverse transcript-polymerase chain reaction (RT-PCR). In accordance to the recommended protocol of the manufacturer, the total RNA was taken from the cultured MC3T3-E1 cells using Trizol reagent (Invitrogen, San Diego, CA). For the synthesis of single-stranded cDNA (cDNA synthesis kit; Invitrogen), the amount of 2 μg of total RNA was employed. The forward 5′- CAAGGGTGAGACAGGGCAAC −3 and the reverse 5′-AACTGGAATCCATCGGT-3′ primers were chosen to amplify type I collagen. The conditions of RT-PCR was 35 cycles of 95 °C for 15 s, 60 °C for 30 s and 72 °C for 60 s.

The cDNA for the housekeeping gene Glyceraldehyde 3-phosphate dehydrogenase (GAPDH) was amplified using specific primers as an internal standard. Through electrophoresis on 2.0 % agarose gel, the RT-PCR products were separated for 30 min. Then using the ultraviolet (UV) light using Electronic UV trans-illuminator (Toyobo Co. Ltd, Tokyo, Japan), the RT-PCR products were visualized after staining them with ethidium bromide.

### Senescence of MC3T3 cells

We performed senescence analyses of the MC3T3 plated on the PTMP1 treated and untreated collagen films by using a beta-galactosidase (β-gal) staining kit (Sigma-Aldrich). MC3T3 were cultured on the collagen film or the denatured collagenase-treated collagen film for 12 h. Thereafter, β-gal staining were performed according to the manufacturer’s protocol. The acquired data were handled with a modified Zeiss Axiovert 200 microscope with a 40x phase-contrast objective lens was used to observe the β-gal stained samples. From the senescence image, we use image processing to determine the percentage of cell with and without β-gal staining indicated by blue and non blue color respectively. Any colors on any images are usually represented by color code of RGB band ranging from 0 to 255 for each color band, with 0 represents black color and 255 represents white color. The RGB value for blue color with β-gal staining in the image ranges from 161 to 162 for R band, 153–187 for G band and 113–162 for B band. The image processing algorithm will calculate the number of pixel covered by the blue spots on the image by counting the total number of pixel that falls between the blue color code. For example, suppose the image processing algorithm detected [0,0,162,0,0,0] in the R band from a given image. This means the total blue colored and non-blue colored pixel in R band is 1 and 5 respectively. In terms of percentage, we have 20 % blue and 80 % non-blue. By repeating this on G and B band and summing all the blue colored pixel, we obtain the percentage of blue from the image and the results are represented on a bar graph.

### Denatured collagen film

The substrates of round cover glasses (diameter = 18 mm, Marlenfeld GmbH & Co. KG, Lauda-Königshofen, Germany) were treated with reactive O_2_ plasma (20 sccm, 200 mTorr, 50 W for 3 min) to immobilize type I collagens on the glass surfaces. The substrate was then cleaned by sonication in toluene solution for 1 min, washed with 70 % ethanol, and dried under nitrogen gas stream. To prepare the collagen with PTMP1 coated glass surface, we diluted 1 mg/mL collagen type I (from rat tails; Invitrogen, Carlsbad, CA, USA) to a final concentration of 0.32 mg/mL and final concentration of 0.32 mg/mL PTMP1 using PBS (pH 7.4) solution, which contained calcium and magnesium; the resulting mixture was incubated overnight with previously cleaned glass at 37 °C. The samples were then washed with PBS solution. Collagenase type I (from Clostridium histolyticum; Sigma-Aldrich) was diluted to a final concentration of 2 mg/mL using PBS solution containing calcium and magnesium. The prepared collagen film surface was treated for 5 min at 37 °C to form denatured collagen films on a glass surface. The collagen and denatured collagen conjugated glass surfaces were immediately washed with PBS solution. For SEM imaging, both collagen films were rinsed with deionized water and dried with nitrogen gas for the analysis of structural properties. The samples were mounted on metal stubs and coated with platinum (SC7640 model, Quorum Technology). SEM images were captured using SEM (JEOL JSM-7401 F, Japan) at an acceleration voltage of 15 kV.

## Results and discussions

### Expression and purification of PTMP1

There had been some previous attempts to express the recombinant mussel proteins in *E. coli* culture but some attempts were met with unsuccessful results probably due to the differences of codon usage, which is the tRNA utilization difference between mussel and *E. coli* [16, 17]. Thus, to ensure that the mussel protein can be successfully expressed in *E. coli*, all the minor codons in PTMP1 gene sequence to *E. coli* were switched to the major codons of *E. coli* [17]. To express PTMP1 in *E. coli*, we cloned the cDNA of PTMP1 with the major codons of *E. coli* was cloned into an expression vector that contains IPTG-inducible T7 promoter and were successful in expressing the recombinant PTMP1 in *E. coli* culture (Fig. 1b). With this redesigning of the gene sequence based on the codon usage preference of *E. coli*, PTMP1 was able to be sufficiently expressed in *E. coli* (~25 % of total cellular protein, Fig. 1b). By analyzing the protein expression through SDS–PAGE depending on time, the protein expression reached its plateau 9 h after the IPTG induction where the cells were the collected and the PTMP1 was purified.

We separated the recombinant PTMP1 into two fractions, i.e., the soluble and the insoluble, and we found that the recombinant PTMP1 was contained in the insoluble fraction. From Fig. 1b, lane IS, we observed that PTMP1 was overexpressed in *E. coli* in the form of insoluble inclusion bodies. Through one-step extraction using 10 % (v/v) acetic acid, PTMP1 was easily purified with a purity of 95 % (using Coomassie blue-stained SDS-PAGE gel image analysis & amino acid analysis) as shown in Fig. 1b, lane A.

PTMP1 has a 15 mol% of positive charged amino acid residue (Table 1), thus it can be easily contaminated especially with the endo-toxins, which are negatively charged biomacromolecules of the *E. coli* cell [18]. This endo-toxin from the cell membrane debris, especially LPS, which is a biomacromolecule consisted of a lipid and a polysaccharide linked by a covalent bond, causes the negative effect to the cell proliferation. Thus, the extracted PTMP1 from the *E. coli* cell debris without removing the endo-toxins can be harmful for biomedical usage and mammalian cell culture experiments.

**Table 1:** Amino acid analysis of PTMP1

Compound Name	Amount [nmoles]	Amount [%]	Theoretical
Asx	25.5	10.7	11.9
Thr	17.9	7.5	5.1
Ser	12.4	5.2	6.8
Glx	17.9	7.5	10.4
Pro	9.0	3.8	5.1
Gly	24.4	10.2	9.7
Ala	18.7	7.8	7.1
Cys	0.9	0.4	1.3
Val	22.5	9.4	9.3
Met	5.5	2.3	2.4
Ile	16.0	6.7	6.2
Leu	17.8	7.4	5.5
Tyr	3.8	1.6	2.2
Phe	11.3	4.7	4.6
His	5.6	2.4	2.0
Lys	22.0	9.2	8.2
Arg	8.4	3.5	2.2
Total	239.7	100.0	100.0

In view of this, the recombinant PTMP1 was further purified with the C-8 reverse phase HPLC as shown in Fig. 1c. The recombinant PTMP1 was eluted in 30 % acetonitrile concentration, which is the same concentration of eluting native mussel foot protein-1 (mfp-1) [19]. We have used amino acid analysis (Table 1) and MALDI-TOF mass spectrometry (Fig. 1d) to confirm that recombinant PTMP1 protein had been successfully purified.

The predicted protein mass of the recombinant PTMP1 based on cDNA deduced sequence of PTMP1 is ~49 kDa. However, through the SDS-PAGE analysis (Fig. 1b), the recombinant PTMP1 has the molecular mass close to 40 kDa, which is 9 kDa smaller than the predicted one. To ascertain the actual molecular mass of the purified recombinant PTMP1, we used MALDI-TOF MS analysis and we found that the molecular mass was 48.5 kDa (Fig. 1d). Our results indicate that we had successfully produced PTMP1 in *E. coli* and purified with a relatively simple method, suggesting that PTMP1 could be mass-produced in *E. coli* system economically. In addition, a high purity of PTMP1 warrants the use of the recombinant PTMP1 for further subsequent experiments and analyses. A highly purified PTMP1 is vital to ensure further results obtained from experiment are attributed exclusively to PTMP1 and not other unknown proteins.

### Cell spreading, proliferation, and adhesion on PTMP1-coated surface

Having successfully obtained the purified PTMP1, we proceeded with the investigation of cell spreading, proliferation and adhesion abilities of PTMP1 with the use of collagen secreting mammalian osteoblast (MC3T3-E1). Comparatives studies were conducted with one surface coated with PTMP1 and another uncoated surface as a negative control. Previous studies have reported that PTMP1 possesses vWF A domain that bears structure similarity as integrin [7]. Integrin plays significant role in mediating cell adhesion, spreading and the formation of actin-filament and integrin is also known to bind with collagen. Thus, to examine the effect of PTMP1 on cell spreading, proliferation and adhesion, we focused on the quantitative analyses of the number of cells that attach to both the coated and uncoated surfaces and qualitative evaluation of the cell-spreading state both on the same surfaces.

In general, cell attachment and spreading could happen in any environments that contain serum regardless of the surface coating. This is due to majority of cell attachment and spreading factors such as fibronectin, vitronectin and cytokines are found in serum. In view of this, all our cell experiments (except for the cell-proliferation assay) were carried out under serum-free condition.

**Fig. 2:**
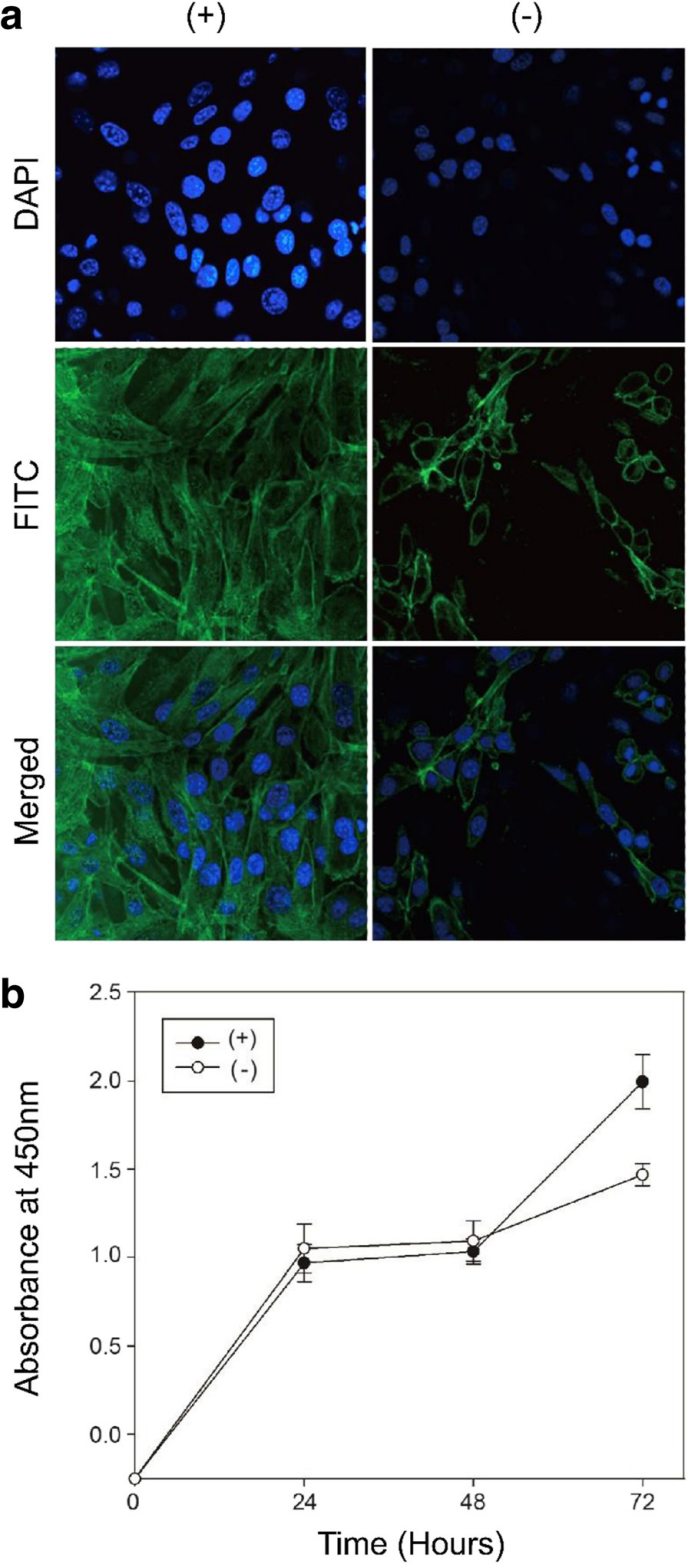
**a** Cell spreading and adhesion and **b** Cell proliferation of MC3T3-E1 cells on PTMP1-coated surface. Each value and error bar is the mean of triplicated sample and its standard deviation. (+) PTMP1 coated tissue culture polystyrene; (−) bare tissue culture polystyrene

By using confocal microscopy, we analyzed the cell spreading through the observation of the morphology of the attached cell on the surfaces (Fig. 2a). Suspended cells that were spread on the coated surface showed a change in shape from round to becoming longer or larger. On the other hand, suspended cells that were spread on the uncoated surface showed the change of shape in the opposite direction, i.e. becoming shorter or smaller. This shows that surface coated with PTMP1 has good cell spreading capability.

As a quantitative measure of the improvement in cell proliferation in the presence of PTMP1, the nucleus stain DAPI clearly shows that in the PTMP1 coated surface, the number of osteoblast cells is approximately 2 times more attached compared to the condition of non PTMP1 coated surface. In terms of cell size, the average maximum stretched length of the osteoblast cell is about 25 nm in the PTMP1 coated surface while this is measured about 10 nm in the non PTMP1 coated surface. The substantial increase in this length coupled with the significant increase in the osteoblast cell number clearly indicates the enhancement of cell proliferation in the presence of PTMP1.

Additionally, the superiority of surface coated with PTMP1 in cell spreading compared to uncoated surface was also exemplified through the observed clear cytoskeleton formation on the latter. The formation of cytoskeleton was observed by actin labeling using FITC-conjugated phalloidin in MC3T3-E1 cells (Fig. 2a). The observations obtained from the confocal microscopy demonstrated that PTMP1 induced actin cytoskeleton formation, which enhance the ability of cells to make focal adhesion. Focal adhesion, which has influence on the morphology, motility and the survival of cells, occurs, when a cell adhesion is mediated by a cell recognition motif through integrin. Thus, with PTMP1 having the vWF -like domain that mediates adhesion, or crosslinks between collagen molecules thereby it could potentially contribute positive effects on cell proliferation.

To investigate the effect of PTMP1 coating on cell proliferation, the CCK-8 assay was performed. In this CCK-8 assay, the measurement of metabolic activity of viable cells and absorbance at 480 nm has a linear correlation with the number of live cells attached to the surfaces. There is a minute difference observed in cell proliferation activity for the first 48 h. The reason for this could be that at the initial stage, cells can competently condition themselves to surfaces by depositing extracellular matrix proteins in the attempt to achieve a good substrate and such conditioning has known to take place [20]. Nevertheless, PTMP1-coated surface did eventually show substantially higher levels of cell proliferation and growth compared to uncoated surface for MC3T3-E1 cells (Fig. 2b) indicating PTMP1 enhance cell proliferation activity.

Our results strongly suggest that the vWF-like protein such as PTMP1 mediated cell adhesion ability that subsequently leads to the improved cell spread and the formation of actin cytoskeleton. A very probable reason for this is the presence of two vWF domains in PTMP1, which may highly contribute to the crosslinking with collagen on the cells. This finding would see recombinant PTMP1 having significant impact on cell culture and tissue engineering. As there are numerous cell adhesion and recognition motifs for each organism or tissue, if any of the particular motif can be fused with PTMP1 (e.g. RGD motif), specific target cells can bind to artificial ECM or biomaterials in a tight manner. Also, the production of several types of specific cell adhesion biomaterials with these specific target cells can be made in a more economical sense owing to the potential mass-production characteristic of PTMP1.

### Collagen expression from and binding with PTMP1 and senescence of ECs

**Fig. 3:**
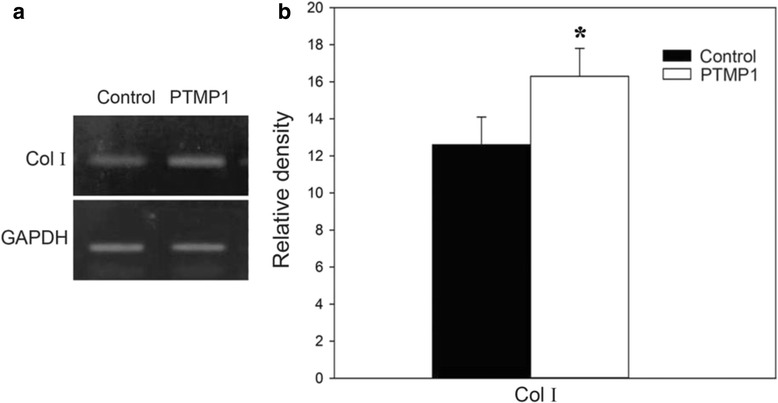
**a** Real Time PCR expression analysis of Col type I of MC3T3-E1 after 3 days on both PTMP1 coated surface (PTMP1) and uncoated surface (NC) **b** Quantification of the band intensity of Col I normalized to GAPDH. Each value and error bar is the mean of triplicated sample and its standard deviation (statistical significance is designated by *p < 0.05)

Type I collagen is the major protein acting as an integral component of the integrin-mediated signaling pathway, and Col I functions as a key marker for activation of integrin mediated signaling that is important for cell proliferation spreading, migration, and survival. Specifically, Col I interacts with the cytoplasmic tail of integrin b subunits through the cytoskeletal proteins, talin and paxillin, and is recruited for focal adhesion [21]. As collagen are expressing via activation of integrin-mediated signaling, which creates a binding site for Src homology 2 (SH2) domain-containing proteins, such as Src family kinases (SFK), phosphatidylinositol 3-kinase (PI3K), and phospholipase Cc (PLCc). Subsequently, SFKs phosphorylate additional tyrosines of the catalytic domain and the carboxy-terminal region of Col I which, in turn, promotes gene expression associated with cell proliferation, spreading, and survival. For direct assessment of Col I expression by integrin signaling in MC3T3-E1 cells on the PTMP1 coated surface, collagen gene expression levels were analyzed using real time PCR (Fig. 3b). The level of collagen gene expression was higher on the PTMP1-coated surface (30 %) compared with GAPDH as the loading control than that of the uncoated surface.

**Fig. 4:**
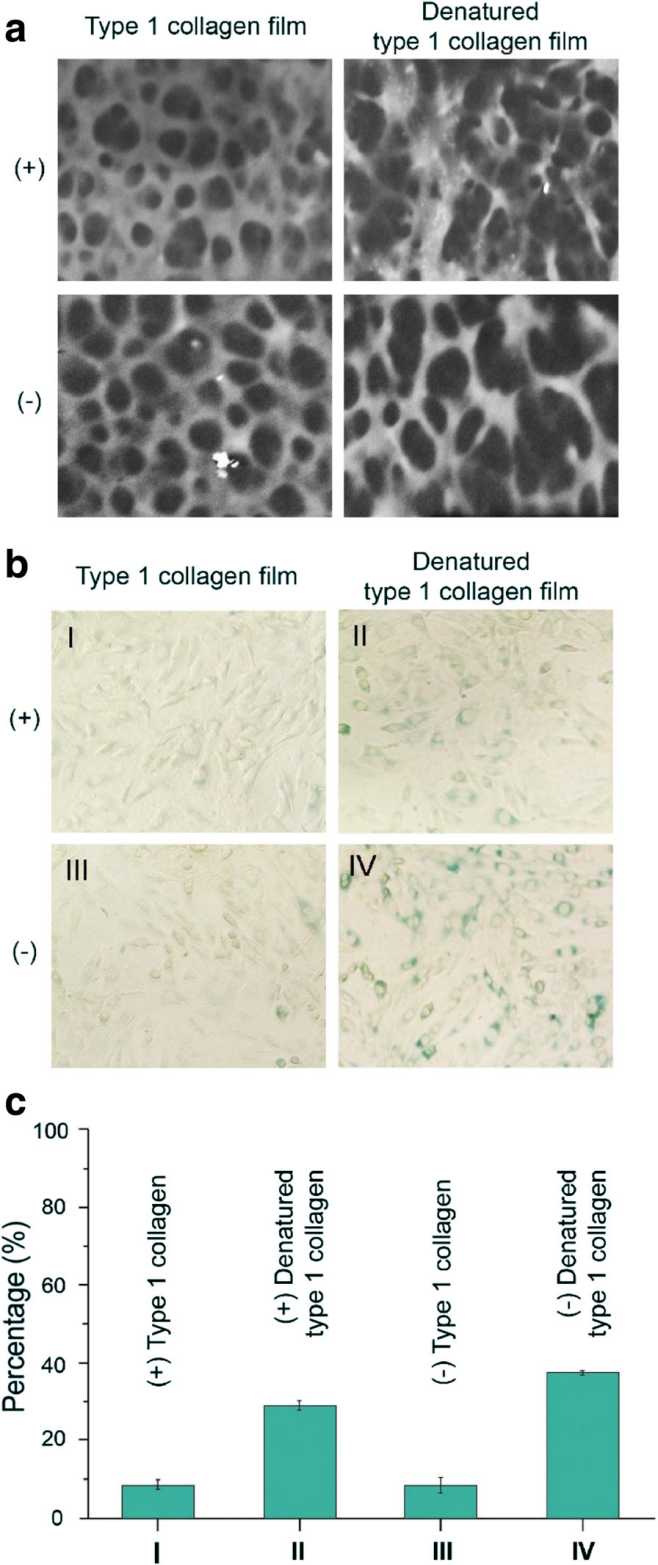
**a** SEM image of type I collagen films in the presence (+) or absence (−) of PTMP1, **b** Senescence of MC3T3-E1 cells on Collagen film and denatured collagen film in the presence (+) or absence (−) of PTMP1. **c** Intensity plot of the senescence in (**b**). The ordinate is the intensity plot in percentage indicating the amount of senescence detected in (**b**). The abscissa is the four conditions shown in (**b**)

As described at the introduction, vWF A domain is found in integrins, which are able to bind with various macromolecular ligands including collagen [8, 10]. Also, it was reported that the mechanical properties are mainly determined by the macromolecular structures of collagen, elastin, and proteoglycans [22, 23]. As PTMP1 has two of these vWFs, it would likely to crosslink with collagen and helps inhibiting the collagen from denaturation. Thus, PTMP1, which is regarding as having a homology of integrins vWF domain, was treated with collagen to prepare collagen film. As shown in Fig. 4a, collagen film was constructed well with or without PTMP1, while denatured collagen films were collapsed. As a quantitative measure, we measured 10 holes of the denatured collagen. These holes are indicated by the dark rough circular area shown in Fig. 4a. The average size of the hole has a diameter of 300 nm ± 17 nm. When we compare this with the presence of PTMP1 on the denatured type 1 collagen film, the average size of the hole reduced by approximately half (140 nm ± 20 nm), indicating the ability of vWF domain of PTMP1 in holding the structure of the collagen.

To study the senescence, fibroblast cultured on the PTMP1 coated surface and noncoacted surface were stained by using a β-gal staining kit and microphotographed with a bright-light microscopy (Fig. 4b). The fibroblast cells on PTMP1 coated surface and uncoated surface were cultured for 6, 24 h, respectively. Osteoblast cells on the PTMP1 uncoated surface (Fig. 4b) in which the clustered cell regions exhibit senescence (blue color). To quantify the amount of senescence on the cell, image processing is used to quantify the intensity of the blue color presence in the cell. The image processing calculates the number of pixel that contains the blue color and returns the value in percentage (Fig. 4c). Especially, the 24 h cultured group has more senescent cells, compared to the groups of 6 h cell culture on PTMP1 coated surface. Our results collectively indicate that collagen is induced following interactions between PTMP1 and integrin of the cell membrane, leading to enhancement of integrin-mediated cell behaviors, such as proliferation, spreading, and survival.

## Conclusions

In summary, we mass produced and purified PTMP1 originated from *M. galloprovicialis* in *E. coli* system in order to look at its possibility as a component for the extracellular matrix (ECM). With a similar homology with von Willebrand factor (vWF), which is a large multimeric glycoprotein and widely distributed in other extracellular protein superfamilies including integrins which are involved in binding various macromolecular ligands, we found that PTMP1 enhanced cell adhesion and contributed positive effects on cell spreading (superior cytoskeleton formation) and proliferation. Also, a recombinant PTMP1 promoted higher collagen expression as well as successfully interacted with collagen, subsequently effected on solid formation of collagen structure and cell viability, which may better mimic the ECM structure. Collectively, it should be noted that PTMP1 played vWF-like roles as mediating cell adhesion, spreading, and proliferation and binding with collagen. We do recognize that while the enhancement and promotion induced by PTMP1 on cell and collagen respectively may not be largely huge, they are still substantial given that PTMP1 is not their natural binding substances. This study suggests that mammalian cell adhesion and marine underwater adhesion have in common and recombinant PTMP1 could be applicable for the development of biomedical adhesives and used on cell culture and tissue engineering.
